# *Haemophilus influenzae* HP1 Bacteriophage Encodes a Lytic Cassette with a Pinholin and a Signal-Arrest-Release Endolysin

**DOI:** 10.3390/ijms21114013

**Published:** 2020-06-04

**Authors:** Monika Adamczyk-Popławska, Zuzanna Tracz-Gaszewska, Przemysław Lasota, Agnieszka Kwiatek, Andrzej Piekarowicz

**Affiliations:** Warsaw University, Faculty of Biology, Institute of Microbiology, Department of Molecular Virology, Miecznikowa 1, 02-096 Warsaw, Poland; zuzannatracz@gmail.com (Z.T.-G.); plasota1@gmail.com (P.L.); akwiat@biol.uw.edu.pl (A.K.); anpiek@yahoo.com (A.P.)

**Keywords:** HP1 phage, lytic system, holing, pinholin, endolysin, signal-arrest-release

## Abstract

HP1 is a temperate bacteriophage, belonging to the *Myoviridae* family and infecting *Haemophilus influenzae* Rd. By in silico analysis and molecular cloning, we characterized *lys* and *hol* gene products, present in the previously proposed lytic module of HP1 phage. The amino acid sequence of the *lys* gene product revealed the presence of signal-arrest-release (SAR) and muraminidase domains, characteristic for some endolysins. HP1 endolysin was able to induce lysis on its own when cloned and expressed in *Escherichia coli*, but the new phage release from infected *H. influenzae* cells was suppressed by inhibition of the secretion (*sec*) pathway. Protein encoded by *hol* gene is a transmembrane protein, with unusual C-out and N-in topology, when overexpressed/activated. Its overexpression in *E. coli* did not allow the formation of large pores (lack of leakage of β-galactosidase), but caused cell death (decrease in viable cell count) without lysis (turbidity remained constant). These data suggest that *lys* gene encodes a SAR-endolysin and that the *hol* gene product is a pinholin. HP1 SAR-endolysin is responsible for cell lysis and HP1 pinholin seems to regulate the cell lysis and the phage progeny release from *H. influenzae* cells, as new phage release from the natural host was inhibited by deletion of the *hol* gene.

## 1. Introduction

Viruses infecting bacteria have different strategies for the releasing of the produced progeny phage from the host cell. Most dsDNA and dsRNA bacteriophages use a holin-endolysin system to lyse their bacterial hosts and release new phage progeny [1]. Among the most widely studied are bacteriophages infecting *Escherichia coli* such as lambda (λ), T7, or phage 21 [1,2,3,4,5]. Currently known lysis systems are encoded by two to five genes, organized in a so-called lytic cassette. Two of the genes code for the holin and the endolysin, that are directly involved in induction of cell lysis. The others gene products play an accessory role [1].

The canonical lytic system of lambdoidal phages, such as λ phage, is composed of 5 proteins. The holin gene of λ phage contains a dual-start motif that allows the expression of two products of different lengths. One acts as an effector (holin), and the other as an inhibitor (antiholin) of the holin protein. Such dual start motif was also found for some other holin genes [1,3,6,7,8]. Holins, controlled by anti-holin proteins, accumulate in the cytoplasmatic membrane without damaging the host cell. At a genetically determined time, the holins disrupt the inner membrane by forming one large hole, thereby allowing endolysins to reach and digest the peptidoglycan [1]. In fact, holins act as regulators of the timing of host cell lysis. The role of endolysins is to destroy the cell wall. Two additional proteins, called spanins, participate in the destruction of the outer cell membrane [9]. Lysis induced by such system is generally very rapid, and is completed within a few seconds [1].

Phage endolysins are generally small globular proteins which act as transglycosidases, lysozymes, amidases, or endopeptidases [1,4,10], but multi-functional endolysins have also been described [11]. Most endolysins do not contain a secretory domain, accumulate in the cytoplasm, and depend on holins to pass the inner membrane and reach the peptidoglycan. In the last decade, a group of phage-encoded endolysins that contain a signal-arrest-release (SAR) domain were described [1,2,12]. The SAR domain initially acts as an export signal domain, which directs the endolysin to the periplasm. In export of the SAR-endolysins is involved the host secretion (*sec*) system [1,2,13]. However, usually SAR-endolysins are not processed by signal proteases and not released in the periplasm but accumulate in inactive, membrane-tethered form. Their activation is correlated with the release of these enzymes from the membrane and the refolding to an active form. The release may be linked to the membrane depolarization under action of the associated holin, called pinholin (for review see [1]). As opposed to large-hole-forming holins, pinholins form small holes that depolarize the inner membrane. Recently, the structure of the lethal pinhole of phage 21 was resolved and holes were <2 nm in diameter [6].

Only few systems encoding SAR-endolysin and pinholin were described. The most studied is the lytic cassette encoded by lambdoid phage 21 specific for *E. coli*, composed of pinholin, antiholin, and SAR-endolysin [2]. In addition, other studies described the existence of lytic cassettes, composed of pinholin and SAR-endolysin, encoded by different phages as ΦKMV, that infects *Pseudomonas aeruginosa* [13], Xfas53 phage infecting *Xylella fastidiosa* [14], coliphage P1 [12] or KBNP1315 phage that infects Avian Pathogenic *E. coli* (APEC) [15]. The regulation of SAR-endolysin release seems to be complex but associated with pinholin. Other possibilities of lytic mechanism certainly exist: for example, LydA pinholin is not essential for P1 phage release [12] and the oenococcal phage fOg44 encodes a SAR-endolysin with a cleavable signal sequence [16].

Seven biologically active dsDNA phages have been described as infecting *Haemophilus influenzae*, which is an obligate commensal of the upper respiratory tract in humans but may also be responsible for upper respiratory tract infections and meningitis [17]. Among them, there is the HP1 phage, that belongs to the *Myoviridae* family. HP1 phage genome (NC_001697.1) has been sequenced and is 32 kbp long, encoding 41 potential proteins in five plausible transcriptional units [18]. The proposed lytic module includes the potential *lys* and *hol* genes, encoding potential HP1 endolysin and holing [18].

In the present study, we characterized empirically the proteins encoded by the potential lytic cassette of *H. influenzae* HP1 bacteriophage. We demonstrated that the HP1 lytic system contains a SAR-endolysin and a pinholin with unusual topology, when activated in the inner membrane. HP1 pinholin seems to participate in control of SAR-endolysin and is necessary to lysis of host cells in the native host.

## 2. Results

### 2.1. In silico Analysis of the HP1 Phage Lysis Cassette Showed Two Putative Lysis Genes

**Figure 1:**
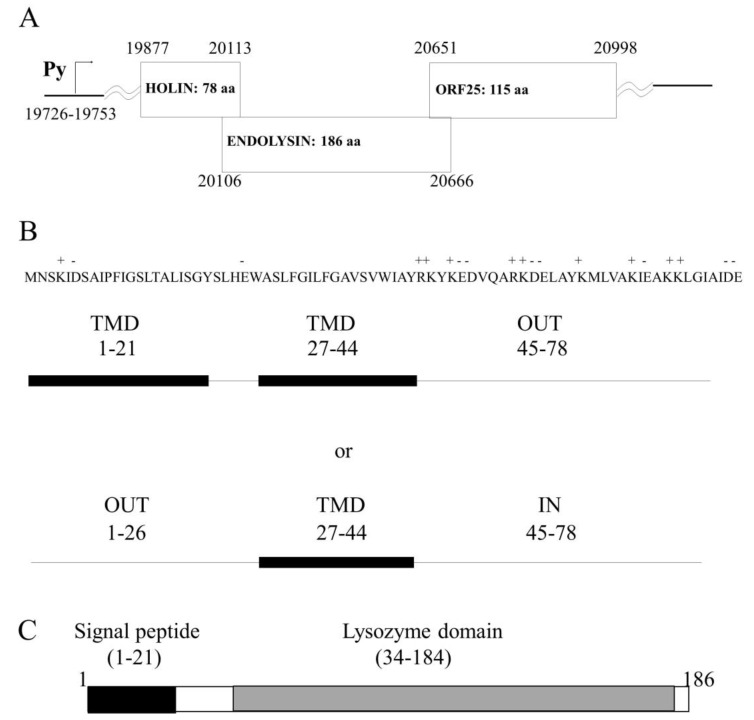
In silico analysis of HP1 phage lytic locus and protein proprieties. (**A**) Organization of lysis *locus* within the genome of HP1 phage. Numbers indicate positions within the genome. (**B**) Amino acid sequence showing charged amino acids of holin protein. Two potential organizations of holin within the cell membrane according to SOSUI (2 trans membrane domains-TMD) and other algorithms (1TMD). (**C**) HP1 phage endolysin domain organization.

The complete nucleotide sequence of the temperate phage HP1 infecting *H. influenzae* was previously determined and the host cell lysis system would be encoded by two open reading frames (ORF), which are transcribed as a late phage transcript [18] (Figure 1A). The *hol* and *lys* genes are located between HP1p29 (ORF24), which encodes for protein showing 100% identity to the tail tube protein of HP2 phage and HP1p32 (ORF25), coding for a protein which has not yet been characterized [18].

Our analysis demonstrates that HP1p32 protein (121 amino acids) shows similarity to LysB protein (Sequence ID: KGQ31227.1) from *Gallibacterium genomospecies* (33% identities, 55% positive). HMMER [19] and TMHMM algorithm analysis revealed the existence of a signal peptide sequence (residues 1 to 25) and a coiled coil region (residues 41 to 93) but no known domain was detected in this protein. HP1p32 cellular localization could not be predicted using in silico algorithms (PSORTb, [20]).

The *hol* gene is the first gene in the lytic cassette and it sequence overlaps by 7 nucleotides the sequence of the gene *lys* (Figure 1A). The analysis of the *hol* gene sequence indicated that the *hol* gene does not present dual start-motif and HP1 phage seems not to encode an antiholin protein. The predicted, encoded protein is 78 aa long. Blast comparison of predicted HP1 holin showed significant homology to a holin-like protein from *Haemophilus quentini* WP_005642949 (96% identities, 98% positive), protein encoded by *Haemophilus haemolyticus* WP_11878507 (95% identities, 96% positive), and holin from *Rodentibacter pneumotropicus* WP_136125977 (74% identities, 87% positive) and also others putative holins, but none of these proteins was characterized empirically in vitro or in vivo. Holins are transmembrane proteins and our analysis with the PSORTb algorithm tool [20] predicted cytoplasmic membrane localization also for HP1 holin. Further, we performed several in silico analysis to predict the potential HP1 holin topology in the bacterial inner membrane. Using the SOSUI algorithm predicting the transmembrane helices [21], we found two transmembrane domains (TMD) for HP1 holin (residues 1 to 21 and 27 to 44) (Figure 1B). The existence of N-terminal TMD was also confirmed by DeepSig [22]. In addition, analysis performed with Phyre2 indicated two TMD [23]. The analysis with other algorithms predicting transmembrane helices (HMMTOP, TMHMM, TMpred, Phobius) indicated that the HP1 holin would have only one TMD (residues 27–44) and that the N-terminal, domain, would be located in the periplasm and the C-terminal domain in the cytoplasm (Figure 1B). Such topology was already proposed by Reddy et al. [24], which predicted that HP1 holin (ID 1.E.7.1.2) is a single-pass membrane protein (TMD formed by 27–45 residues) and belongs to the type II holin superfamily (Transporter Classification Database (TCDB); www.tcdb.org [24]).

The HP1 endolysin was predicted to be encoded by the *lys* gene. The enzyme consists of 186 amino acid residues, and is highly homologous to the lysozymes, encoded by different bacteria and among them to lysozymes encoded by phages (96% identity with *Haemophilus* phage HP2 Lys protein NP_536831.1; 49% identity with *Enterobacteria* phage P1 Lyz protein 1XJU_A, 48% with *Pasteurella* phage F108 Lys protein AAZ93664.1, and 37% with endolysin of bacteriophage K139 AAL47527.1, which infects *Vibrio cholerae*). Only one conserved domain of endolysin-autolysin (phage-related lysozyme and muraminidase-COG3772) was found from residue 34 to 184 (Figure 1C). The SOSUI algorithm predicted a soluble protein. HMMTOP and TMHMM algorithms predicted an N terminal alpha-helix (6–22 residues). The analysis on the N-terminal sequence revealed a stretch of 16 uncharged residues. Such amino terminal extensions typically function as unprocessed transmembrane helices or as cleaved signal sequences for interaction with the secretion machinery [12,25]. As suggested by the SignalP [26] algorithm, this α-helix would be a part of the signal peptide domain (residues 1–22) and its presence would enhance the insertion of the signal peptide into the phospholipid double layer (Figure 1C). These predictions were also confirmed by iPSORT (residues 1 to 30) and by HMMER (signal peptide residues 1 to 24 and lysozyme domain: residues 60 to 178). All analyses suggested that HP1 endolysin is a SAR (signal-arrest-release) endolysin and has lysozyme activity.

### 2.2. Overexpressed HP1 Holin Has C-out, N-in Topology in Bacterial Inner Cell Membrane

Since the in silico analysis was not conclusive, we decided to resolve experimentally the issue of the topology of HP1 holin in the bacterial inner membrane.

For this task, we constructed two plasmids on the basis of pET28a(+) vector with His-tag fused to the N- (pNHisHol) or to the C-terminus (pCHisHol) of HP1 holin.

From *E. coli* BL21(DE3) cultures carrying pNHisHol or pCHisHol constructs, we prepared inverted membrane vesicles (IMV, exposure of intracellular terminus outside) or spheroplasts (exposure of extracellular terminus outside). C- or N-terminus present on the external surfaces of these structures were proteolyzed by adding proteinase K. Then, membrane-associated peptides were isolated and separated by SDS-PAGE. Next, Western blotting, with specific antibodies directed to His-tag, was performed to determine which variant of holin, with His-tagged C- or N-terminus, remained in proteinase-treated IMV or spheroplasts.

**Figure 2:**
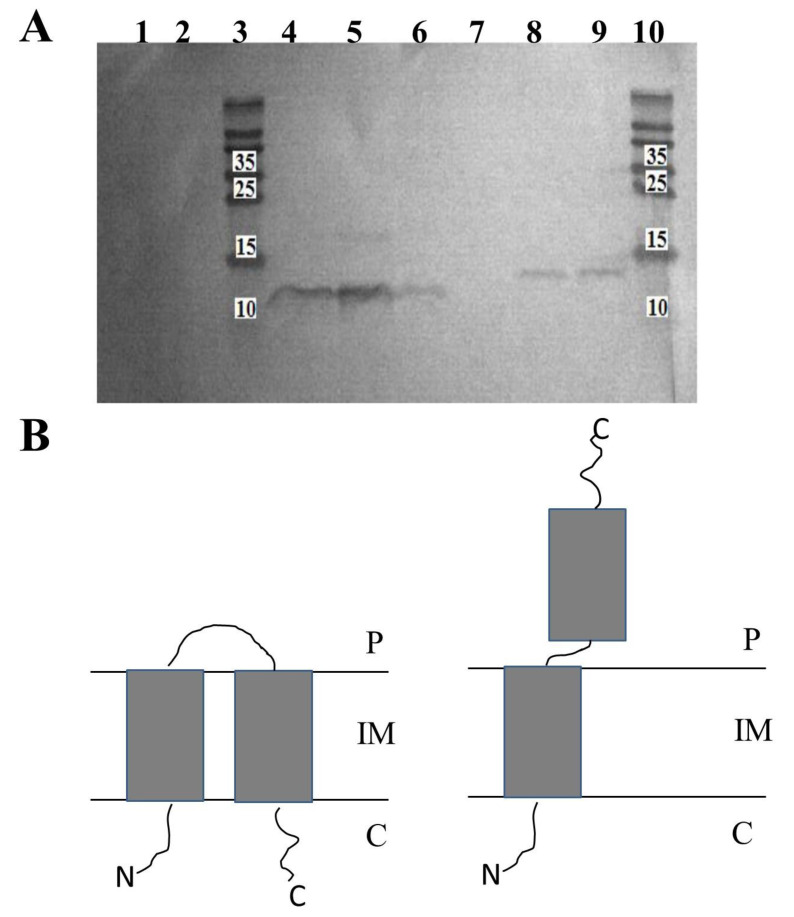
Topology of HP1 holin in the inner membrane of *E. coli* (**A**) Western blot analysis of membrane-embedded proteins isolated from inverted membrane vesicles (IMV) prepared from *E. coli* BL21(DE3) expressing His-tagged holin protein from HP1 phage. When indicated, the peptidyl termini present outside IMV were removed by proteinase K treatment before the extraction of the membrane-embedded protein. Membrane proteins extracted from *E. coli* BL21(DE3) cells carrying control pCHisHol without induction with IPTG (line 1) or control pNHisHol without induction with IPTG (line 2); Membrane proteins extracted from *E. coli* BL21(DE3) cells carrying pCHisHol extracted from: (line 4) IMV treated with proteinase K, (line 5) IMV without the proteinase K treatment, (line 6) whole cells without proteinase K treatment. Membrane proteins extracted from *E. coli* BL21(DE3) cells carrying pNHisHol extracted from: (line 7) IMV treated with proteinase K, (line 8) IMV without the proteinase K treatment, (line 9) whole cells without proteinase K treatment. Lines 3 and 10: Protein Ladder PageRuler^TM^ (ThermoFisher). Numbers represent molecular weight of ladder proteins in Kda (**B**) Schematic model of HP1 holin (P: periplasm, C: cytoplasm, IM: inner membrane). Up: the inactive, non-lethal form, down: the active-lethal form (experimentally confirmed). The exact activation mechanism of holin from non-lethal to lethal form is unknown.

As shown in Figure 2, the detection of His-tagged termini was successful only in case of induction of N-His-tagged or C-His-tagged holin expression by IPTG (Figure 2A, lines 6 and 9, respectively). In extracts prepared from non-induced cells (negative control), His-tagged holins were not detected (Figure 2A, lines 1 and 2). Going further, we have detected C-terminal His-tag of HP1 holin in protein extracts from IMV, even after the treatment with proteinase K (Figure 2A, lines 4 and 5). As the IMV surface represents the cytoplasmatic surface of the cell membrane, the observation that C-terminus is protected from the proteinase K activity indicated that C-terminus of HP1 holin is oriented to the periplasm. On contrary, the His-tagged N-termini were not detected in the samples of membrane proteins isolated from IMV treated with proteinase K (Figure 2A, line 7), whereas such detection was possible in the case of IMV untreated with proteinase K (Figure 2A, line 8) or membrane proteins from whole cell lysates (Figure 2A line 9). These results indicate that N-terminus of the HP1 holin is located on the cytoplasmic side of the cell membrane (sensible to proteinase K treatment in IMV).

Spheroplasts are structures formed only by inner membrane. Preparation of spheroplasts from *E. coli* cells expressing His-tagged holins was unsuccessful. We hypothesize that the inner membrane was in this condition too damaged to allow the preparation of spheroplasts and detection of His-tagged holins.

**Figure A1:**
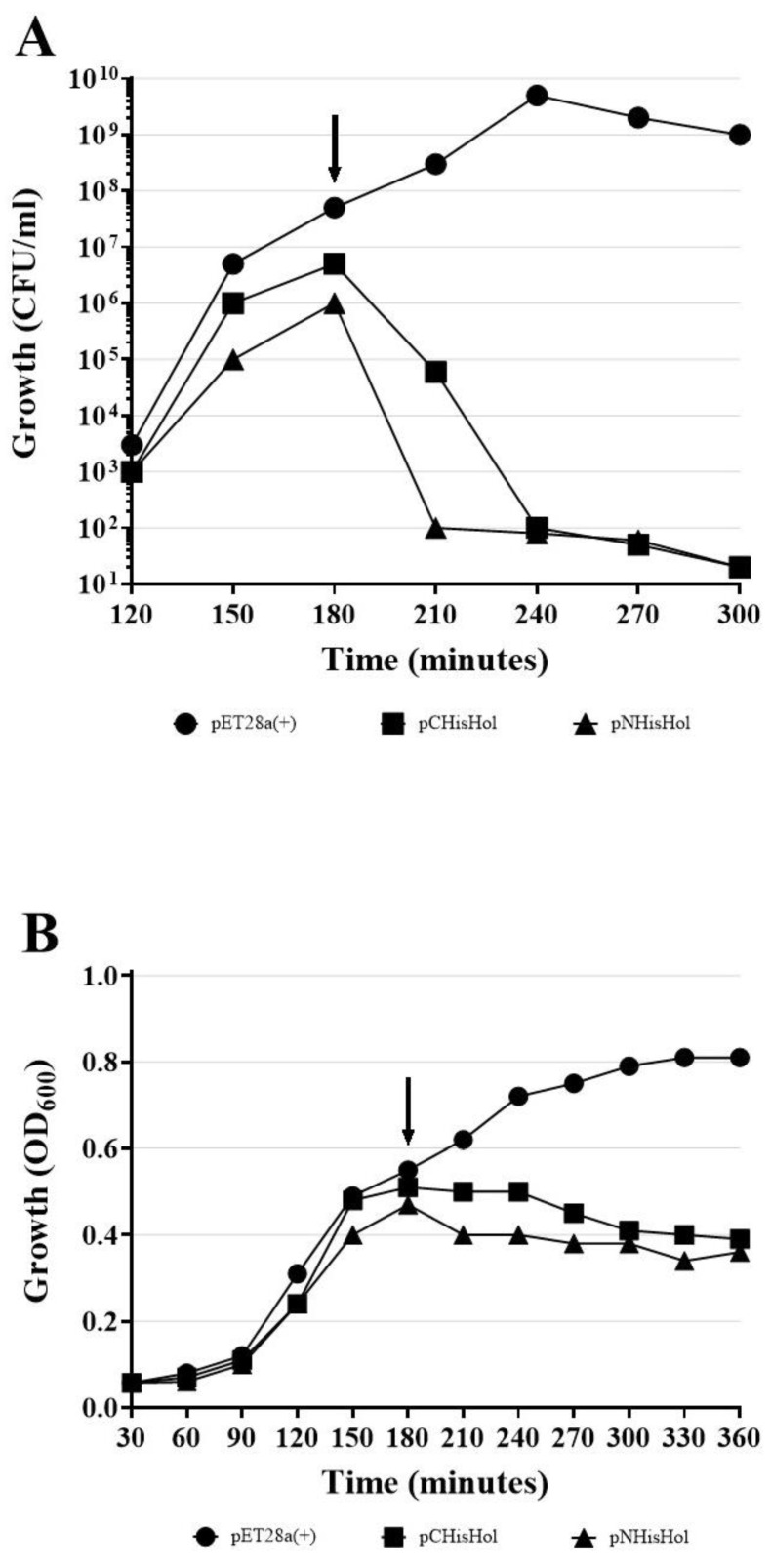
Growth of *E. coli* cells carrying pET28a(+), pCHisHol and pNHisHol. (**A**) Viable cells count (CFU/mL). (**B**) Culture turbidity OD_600_. Induction of lytic genes expression was performed by addition of IPTG (black arrows). Results are representative of three independent experiments.

We also verified, that the addition of His-tag did not affect the HP1 holin conformation, since His-tagged HP1 holins induced cell death, as observed by colony-forming units (CFU/ ml) decrease after induction of expression without cell lysis (OD_600_ of induced cultures remained constant), when overexpressed in *E. coli* (Figure A1) on the same way as wt HP1 holin did (see Section 2.6).

All these data together demonstrated that HP1 holin is a transmembrane protein with C-terminus directed to the periplasm and N-terminus placed on the cytoplasmic side of the *E. coli* cell membrane when overexpressed/activated (Figure 2B).

### 2.3. Inactivation of hol Gene Blocks the Lysis of H. influenzae Cells by HP1 Phage and Prevents the Release of New Phage Particles

**Figure 3:**
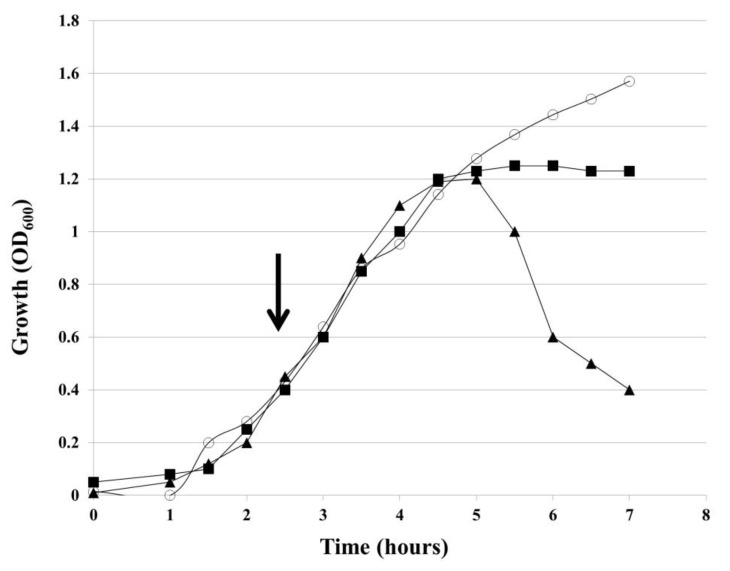
Mutation in the *hol* gene prevents the lysis of *H. influenzae* cells by HP1 phage in vivo. *H. influenzae* Rd30 cells, non-lysogenized (○) or lysogenized with wt HP1 phage (▲) or HP1Δ*hol* phage (■) were growth until exponential phase and phage lytic cycle was induced by addition of mitomycin C (black arrow). The culture turbidity (OD_600_) was checked every 30 min. Results are representative of three independent experiments.

*H. influenzae* Rd30 cells, lysogenized with wild-type HP1 (wt HP1) or *hol*-deficient HP1 phage (HP1Δ*hol*), were grown in liquid culture and OD_600_ of the culture was measured every 30 min. After OD_600_ of lysogenic bacteria reached ~0.4, mitomycin C was added in order to induce the prophage excision and to start the lytic cycle. As shown in Figure 3, *H. influenzae* (wt HP1) cells began to lyse 2.5 h after induction of prophages by mitomycin C, *H. influenzae* (wt HP1). After reaching the peak of 1.2, OD_600_ dropped to 0.4 within 5 h after induction. In contrast, HP1Δ*hol*-lysogenized cells continued growing and reached a plateau 2.5 h after the addition of mitomycin C. Such an observation suggests that the product of the *hol* gene is necessary for the release of new HP1 phage progeny from host bacterial cells.

**Figure 4:**
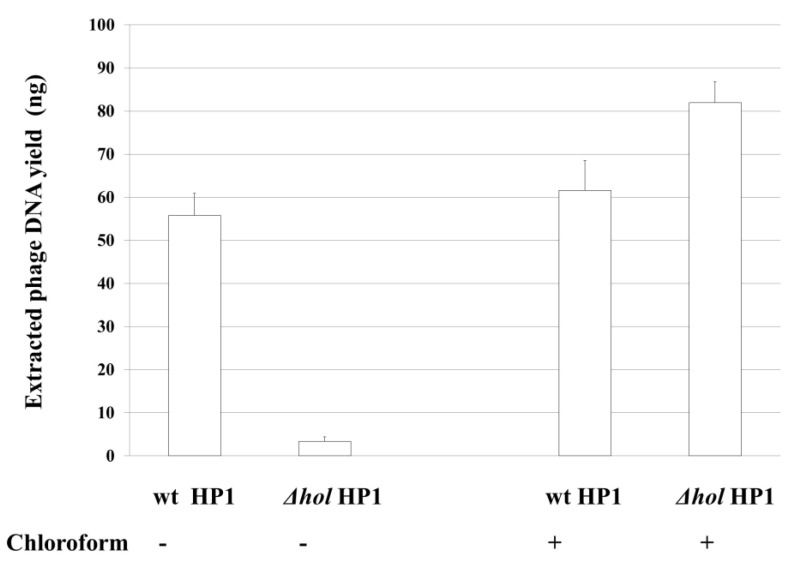
Yield of genomic phage DNA. DNA was isolated from phages released into supernatants after mitomycin C induction of HP1 or HP1Δ*hol* prophages in *H. influenzae* lysogens, compared to those released by chloroform-induced lysis of bacterial cells. wt: wild type HP1 phage, *Δhol* HP1 with inactivated *hol* gene.

Next, we isolated phage particles from culture supernatants and after removing of bacterial DNA and RNA, we performed phage DNA extraction from bacteriophage capsids. As shown in Figure 4, 55.75 (±5.20) ng of phage DNA was isolated from 50 mL of initial culture of induced *H. influenzae* Rd30 (wt HP1) cells, while only 3.4 (±1.00) ng of phage DNA was isolated in the case of cells lysogenized with *hol*-deficient HP1 bacteriophage. As chloroform induces bacterial lysis without affecting the bacteriophage structure, we also extract phage DNA from both cultures treated after chloroform treatment. As shown in Figure 4, the extracted phage DNA from HP1Δ*hol*-lysogenized cells reached in this case 82 (±4.8) ng, while from wt HP1 we have isolated 61.6 (±6.9) ng. So, the absence of lysis of cells lysogenized by HP1Δ*hol* phage (Figure 3) is associated with extremely low phage release into culture supernatants as demonstrated by phage DNA extraction from liberated phages. HP1Δ*hol* phages may be liberated by host lysis with chloroform.

Thus, the deletion of the *hol* gene and absence of HP1 holin prevented the decrease of culture turbidity and the effective release of new bacteriophage particles from mitomycin C-induced *H. influenzae* (HP1*Δhol*). HP1 holin is indispensable in liberation of HP1 bacteriophage from *H. influenzae* cells.

### 2.4. HP1 Holin Expression during Lytic Cycle of HP1 Phage Causes Reduction in H. influenzae Viability

**Figure 5:**
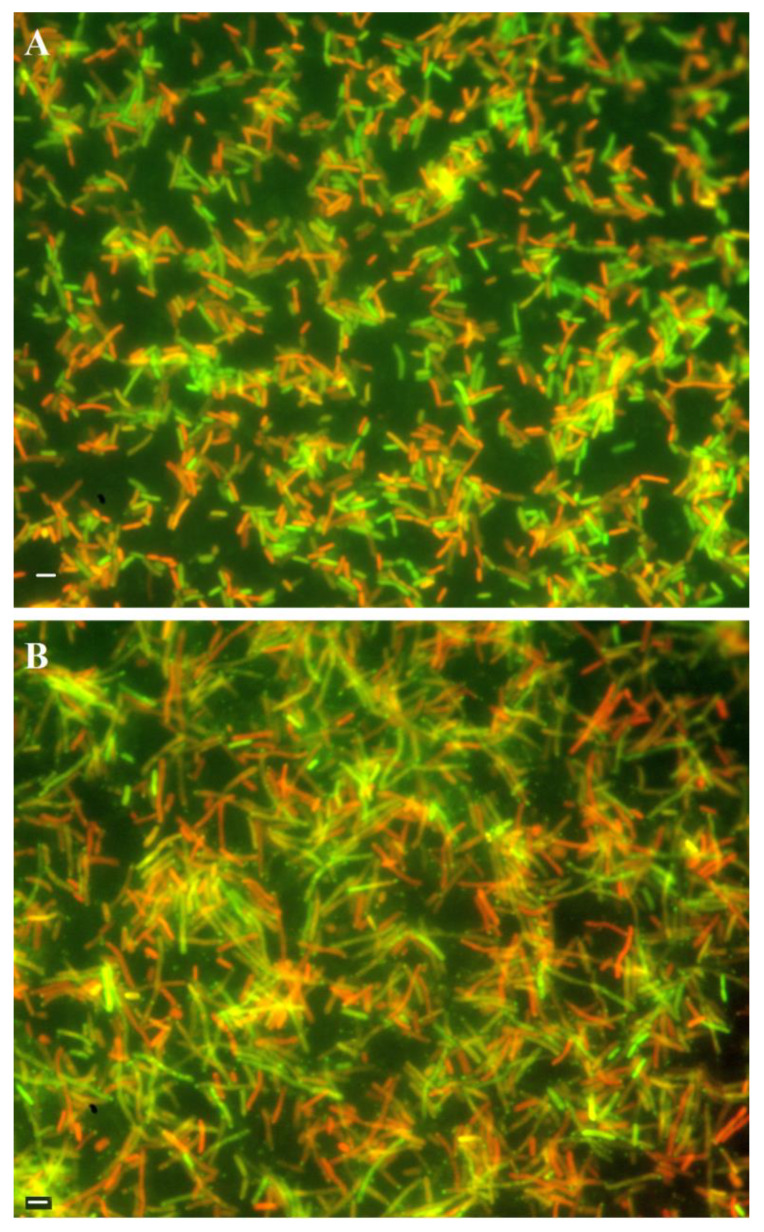
LIVE/DEAD staining of *H. influenzae* cells lysogenized with wt HP1 or HP1Δ*hol* and treated with mitomycin C. The LIVE/DEAD BacLight Bacterial Viability Kit was used for monitoring the viability of bacterial populations as a function of the membrane integrity of the cell. (**A**) Cells lysogenized with wt HP1 phage, (**B**) cells lysogenized with HP1Δ*hol* phage. Bars represent 1 µm. Cells with a compromised membrane that are considered to be dead or dying stain red, whereas cells with an intact membrane stain green.

Bacteriophage lytic cycle was induced with mitomycin C for 2.5 h (Figure 3) in wt HP1 or HP1Δ*hol*-lysogenized *H. influenzae*. At this time, the difference in OD_600_ between wt HP1 and HP1Δ*hol*-lysogenized *H. influenzae* strains is 0.2 (Figure 3). Cell viability was assessed using the LIVE/DEAD bacterial viability kit and the same number of cells was taken for the staining. As shown in Figure 5A, in case of cells with induced wt HP1 replication, the number of dead red-stained cells was lower to this of alive green-stained cells (ratio ~0.8:1) as estimated by ImageJ [27]. In case of cells with induced HP1Δ*hol* phage replication cycle, cells were almost twice more elongated than induced *H. influenzae* cells lysogenized by wt HP1 phage and despite the same number of cells taken for staining, this culture seemed more dense. HP1Δ*hol* phage-containing bacterial cells were not uniformly stained: at the edge of viable bacteria a point with accumulated green stain was observed. In case of HP1Δ*hol* induction, the estimated ratio between dead and alive cells seems to be lower (~0.46:1) than in case of wt HP1 phage, indicating that less cells died in case of replication of HP1Δ*hol* phage than in case of wt HP1.

### 2.5. Membrane Lesions Induced by HP1 Holin Does not Allow the β-Galactosidase Leakage from E. coli Cells

**Figure 6:**
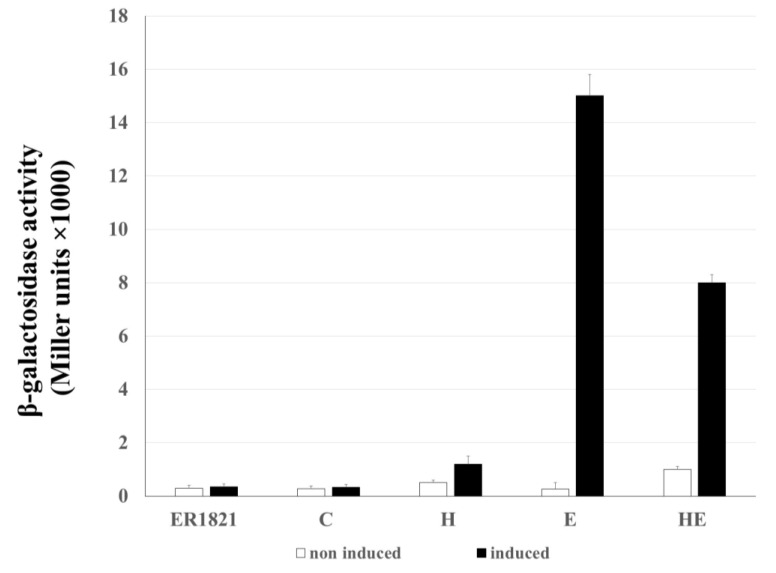
Release of β-galactosidase from *E. coli* ER1821 cells expressing cloned bacteriophage HP1 holin and endolysin. Activity of β-galactosidase was determined in supernatants of cultures. Uninduced cultures were supplemented with glucose, induced cultures with arabinose. C-control ER1821 cells with vector pMPMT4Ω, H-holin expressing cells, E-endolysin expressing cells, HE-holin and lysin expressing cells. Results are average of triplicate experiments.

The damage of the cytoplasmic membrane by lytic proteins was described to be sufficient to allow the leakage of cytoplasmic content, including large proteins like β-galactosidase [28]. The ability of HP1 lytic proteins, encoded by genes *hol* and *lys*, in induction of lesion in host bacterial envelope was investigated by leakage of β-galactosidase from *E. coli* cells (Figure 6). For this, three constructs designated as H (pMPMT4::*hol*), E (pMPMT4::*lys*), and HE (pMPMT4::*hollys*)*,* respectively were introduced to *E. coli* ER1821 cells, naturally expressing β-galactosidase. As control (C), *E. coli* ER1821 carrying empty pMPMT4Ω vector were used, as well as cells without any vector.

Our results show that the increase in extracellular enzymatic activity of β-galactosidase was not observed when *hol* gene expression was induced: the lesions to the membrane made by holin alone are not large enough to allow the passage of large proteins such as β-galactosidase. The induction of the *lys* gene alone induced statistically significantly β-galactosidase activity (15,000 ± 800 Miller units, *p* < 0.01) in the culture supernatant, indicating β-galactosidase leakage through bacterial envelopes. The β-galactosidase activity increased 60 times as compared to uninduced cells coding the *lys* gene (*p* < 0.01). Thus, the overexpression of endolysin alone is sufficient to cause cell lysis even in absence of the HP1 holin.

When *hol* and *lys* genes were co-expressed, the amount of extracellularly released β-galactosidase was 1.875 lower (8000 ± 300 Miller units), than in the case of cells with the *lys* gene expressed alone (*p* < 0.01) (Figure 6).

### 2.6. Expression of hol and lys Genes of HP1 Phage is Lethal to E. coli Cells

To test whether the expression of *hol* or *lys* genes by itself induces bacterial cells lysis, three constructs previously mentioned constructs: H (pMPMT4::*hol*), E (pMPMT4::*lys*), and HE (pMPMT4::*hollys*) were introduced to *E. coli* Top10 cells, which allow precise control of pBad promotor. As control (C), *E. coli* Top10 carrying empty pMPMT4Ω vector were used. We evaluated *E. coli* cell lysis by the measurement of OD_600_ and overall cell death by counting CFU/mL after induction of lytic genes by arabinose addition.

**Figure 7:**
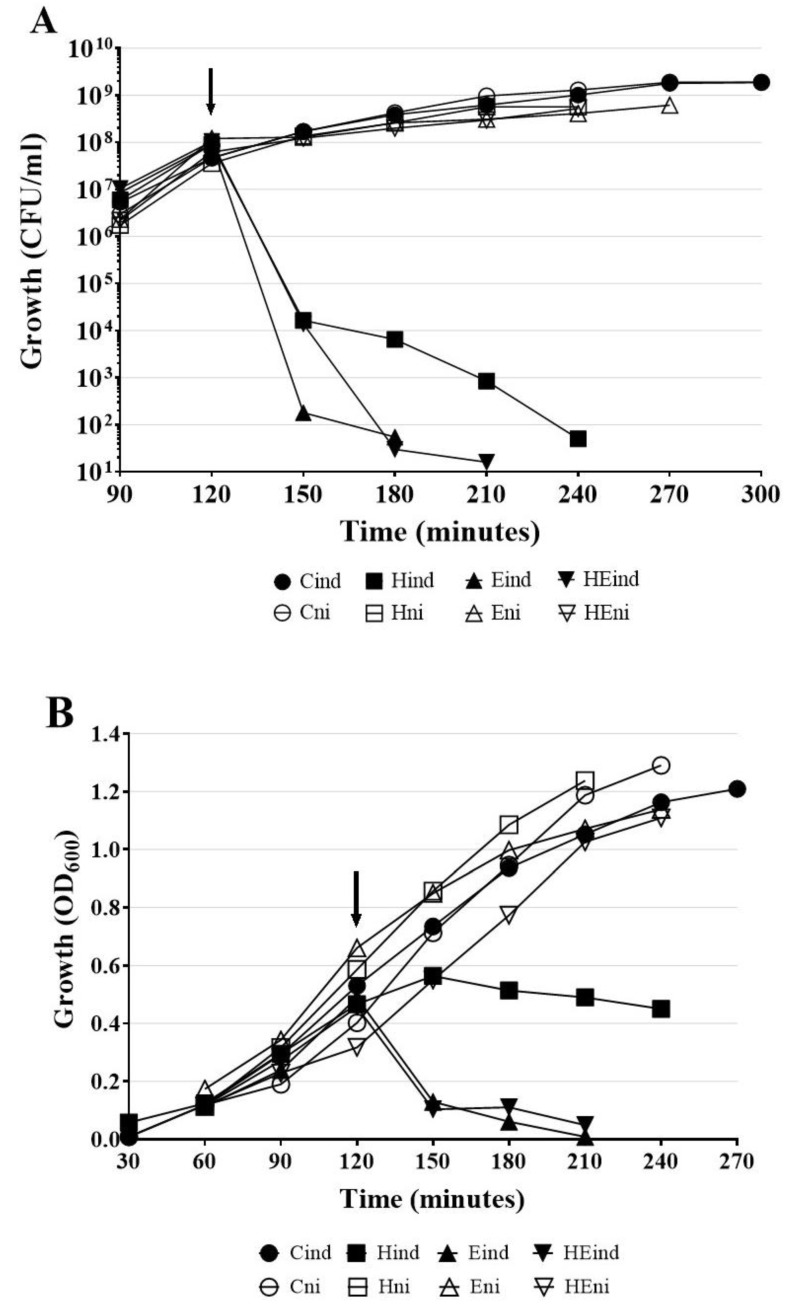
Growth of *E. coli* cells carrying pMPMT4Ω with cloned HP1 lytic genes: holin (H), HP1 endolysin (E), or both (HE) with and without induction with arabinose. (**A**) Viable cells count (CFU/mL). (**B**) Culture turbidity OD600. Induction of lytic genes expression with arabinose took place after 120 min of growth (black arrow). H-cells carrying *hol* gene, induced (Hind) or not (Hni) with arabinose. E-cells carrying *lys* gene, induced (Eind) or not (Eni) with arabinose. HE-cells carrying both lytic genes *lys* and *hol*, induced (HEind) or not (HEni) with arabinose. C-control cells carrying empty plasmid, induced (Cind) or not (Cni) with arabinose. Results are representative of three independent experiments.

The lethal effect of the induced expression of HP1 lytic genes was already observed 30 min after the addition of arabinose (Figure 7A,B, curves Hin, Ein, and HEin). In contrary, the non-induced cells remained viable for at least 3 h. Indeed, viable cells counts showed that after induction of *hol* gene expression, the number of viable HP1 holin-expressing cells dramatically decreased from 9.1 × 10^7^ CFU/mL at induction moment to 6.5 × 10^3^ CFU/mL after 1 h and then to 5 × 10^1^ CFU/mL after 2 h from induction (Figure 7A). The measurement of liquid culture turbidity of *E. coli* Top10 cells expressing the *hol* gen product showed that OD_600_ was constant at 0.450 within 2 h, after the induction of HP1 holin expression (Figure 7B). This suggests that, in absence of other phage proteins, HP1 holin induced bacterial cell death but without causing cell lysis. When synthesis of the *lys* gen product was induced in *E. coli* Top10 cells, the number of viable cells in culture began to decrease even prior to induction. In 30 min from induction, the number of viable cells decreased from 9.15 × 10^7^ to 1.8 × 10^2^ CFU/mL and 1 h after induction the cell number dropped to 5.5 × 10^1^ CFU/mL (Figure 7A). During this time, the turbidity of the culture expressing the *lys* gene also dramatically dropped from 0.48 (induction with arabinose time point) to 0.006, 1 h after induction (Figure 7B). The induction of the *lys* gene expression and synthesis of endolysin cause both, cell death (decrease in CFU/mL) and lysis (decrease of turbidity of the culture) in *E. coli* cells. The above observations are consistent with the results obtained from the study of β-galactosidase leakage after induction of *lys* gene expression (Figure 6). *E. coli* cells with induced expression of both *hol* and *lys* genes behaved similarly to cells carrying only the *lys* gene (Figure 7A,B). Within 60 min after induction, the number of viable HE plasmid-containing cells decreased from 1.04 × 10^8^ to 3.0 × 10^1^ CFU/mL. (Figure 7A). The OD_600_ also dropped from 0.456 to 0.11 during this time (Figure 7B), while the OD_600_ of control uninduced cells (HEni) raised from 0.340 to 0.77 during the same laps of time.

All control cultures carrying cloned genes, which expression was not induced by arabinose, had a similar growth profile as measured by viable cells counting (CFU/mL) or spectrophotometry (Figure 7A,B, curves Hni, Eni, HEni). Control cells (C) containing only pMPMT4Ω vector maintained the growth rate characteristic for *E. coli* (Figure 7A,B). The number of cells increased exponentially and reached 1.88 × 10^9^ CFU/mL (OD_600_ = 1.3) 3 h after arabinose administration.

*E. coli* Top 10 cells carrying three constructs HP1 with lysis genes were also grown in the presence of 1% glucose to stop the expression from the pBad promoter. In this case, no decrease in turbidity or in CFU was observed: the growth was similar to control uninduced cultures (presented on Figure 7A,B), indicating that no lysis occurred when the expression of cloned genes was blocked.

### 2.7. HP1-Induced Cell Lysis and New Phage Release Involve H. influenzae Sec System

**Figure 8:**
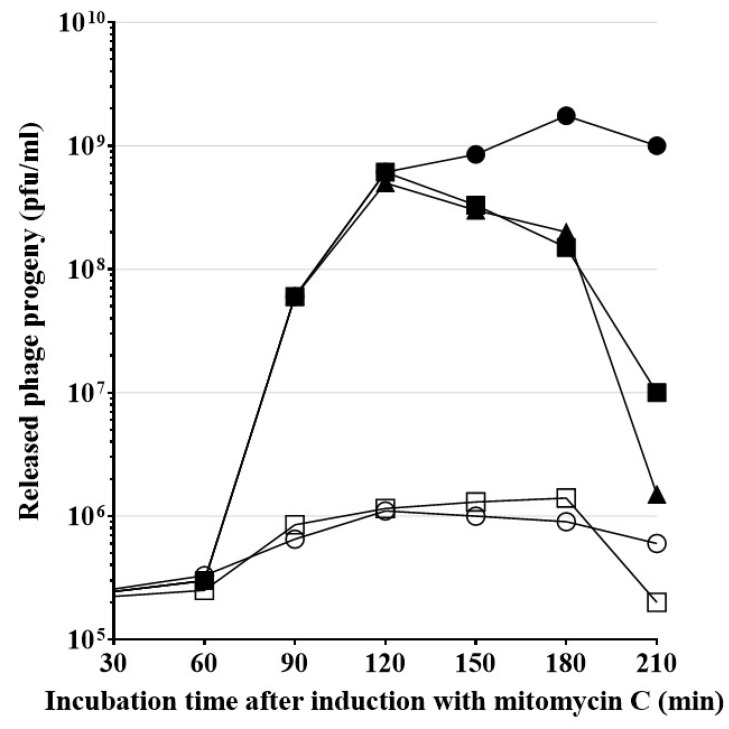
Inhibition of the *sec* system and the phage HP1 release by sodium azide in lysogenic *H. influenzae* Rd30 (HP1) induced with mitomycin C. Cells were grown to an OD_600_ of 0.4 and mitomycin C was then added (time 0). Then, 1 mM sodium azide was added once, at time 0 or 60 or 90 or 120 min after induction and the phage titer was then measured by the double layer agar method for plaque assay. (○) Sodium azide added together with mitomycin C, at time 0; (□) Sodium azide added 60 min after induction; (▲) Sodium azide added 90 min after induction; (■) Sodium azide added 120 min after induction; and (●) control culture, induced with mitomycin C but not treated with sodium azide. Results are representative of three independent experiments.

**Figure A2:**
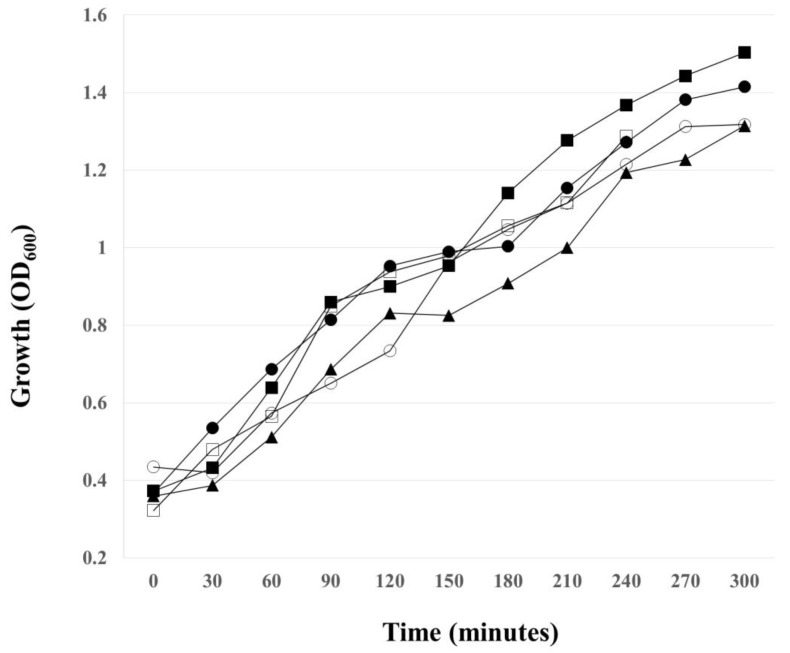
Effect of sodium azide on the growth of *H. influenzae* cells lysogenized with HP1 phage. Then, 1 mM sodium azide was added at time 0 or 60 or 90 or 120 min after bacteria reached OD_600_ ~0.4 (time 0 on the graph) and the culture turbidity was then followed as a function of time. (○) Sodium azide added at time 0; (□) Sodium azide added 60 min after time 0; (▲) Sodium azide added 90 min after after time 0; (■) Sodium azide added 120 min after time 0; and (●) control culture, not treated with sodium azide. Results are representative of three independent experiments.

Endolysins, which contain an SAR sequence, are exported in a membrane-bound state by the host *sec* system, and subsequently are released in a soluble state into the periplasm. Thus, the release of HP1 phage particles from bacterial cells is presumed to involve the transport of the phage endolysin to the periplasm by the *H. influenzae sec* system [4]. Sodium azide (NaN_3_) is the well-known inhibitor of the SecA ATPase activity, necessary for the translocation of exported protein across the membrane [13,29]. As we mentioned before, our predictions indicated that HP1 endolysin contains a SAR sequence. To study the potential involvement of the *sec* system in HP1-induced lysis of the bacterial host, we used 1 mM NaN_3_ to inhibit the HP1 phage progeny release from bacterial cells. Such concentration of sodium azide was not toxic to *H. influenzae* cells, when added to cell cultures (Figure A2). Lysogenic *H. influenzae* Rd30 (wt HP1) culture was treated with mitomycin C at OD_600_ = 0.4, to induce the lytic cycle of the virus. As shown in Figure 8, approximatively 60 min after induction, phage particles were released and counted as plaque-forming units (PFU/mL) in plaque assay. The peak of new phages release occurred 3–4 h after induction with mitomycin C and reached the level of 1.75 × 10^9^ PFU/mL. The inhibition of the *sec* system with 1 mM NaN_3_ blocked the release of phage particles from mitomycin C-induced lysogenic *H. influenzae* Rd30 (wt HP1) cells. As shown in Figure 8, simultaneous addition of NaN_3_ and mitomycin C or adding NaN_3_, 60 min after induction, dramatically stopped the HP1 progeny release: the maximum of release was only about 2 × 10^5^ and 6 × 10^5^ PFU/mL, respectively. Addition of NaN_3_ 90 or 120 min after induction, first slowed the release of new phages, then their number declined rapidly. In this case, the endolysin, exported to periplasm by the *sec* system before NaN_3_ addition, ensured the release of new phage particles, but after addition of the *sec* system inhibitor, no new endolysin is exported and released phage number was stopped.

Since inhibition of the *sec* system decreased the yield of release of new phage particles, our results strongly suggest that the *sec* system is involved in the HP1 phage release from the host *H. influenzae* cells, probably by participating in the transport of SAR-endolysin from the inner membrane to periplasm.

## 3. Discussion

Bacteriophage-induced lysis may be the source of released genetic information accessible for naturally competent bacteria, which are able to uptake foreign DNA from environment. Thus, phage-induced lysis participates in the horizontal gene transfer. Moreover, the current crisis of antibiotic-based treatments calls for alternative strategies for fighting bacterial infections and phage lytic proteins seem to be potential new antibacterial agents. In this context, it is not surprising that the mechanisms by which bacteriophages cause cell death and lysis are extensively investigated.

In this paper, we described the lytic cassette encoded by *H. influenzae*-specific bacteriophage HP1. In addition to *hol* and *lys* genes, our in silico analysis of the whole HP1 genome reveals a third potential gene that may compose the HP1 lytic cassette: ORF25. Indeed, ORF25-encoded protein displays some similarity to LysB from *G. genomospecies*. LysB is annotated as phage lysis regulatory protein and may be a spanin. However, our analysis of ORF25 protein did not reveal any structure similar to known spanins [30].

Our topological predictions demonstrated that the *lys* product is an SAR-endolysin. Overexpression of HP1 endolysin alone in *E. coli* cells caused both, cell death and lysis, as demonstrated by the dropping number of CFU/mL and turbidity of cell culture. Moreover, we demonstrated that the release of HP1 phage particles from induced *H. influenzae* cells (natural host) can be restrained by inhibiting the host *sec* system with sodium azide, the SecA-inhibitor. Such an observation was also made for SAR-endolysins, encoded by different phages: SAR-endolysins are able to induce bacterial lysis without holin presence. For example, introduction of fOg44 lysin, Lys44, gene into *E. coli* BL21(DE3) (pLysS) cells results in very unstable clones even in the absence of IPTG. This instability was due to lethal consequences of Lys44 lysin expression [16]. It was also demonstrated that in the case of phage P1, a certain quantity of P1 SAR-endolysin spontaneously passed into periplasm and was active, without participation of holins [31,32]. In addition, the overexpression of SAR-endolysin from phage KNBP1315 induced the decline in the turbidity of *E. coli* BL21(DE3) [15]. Our data support that HP1 endolysin, like other SAR-endolysins, may access the peptidoglycan in the absence of holin and induce cell death and lysis. However, our results indicated that the lysis induced by SAR-endolysin alone occurred only in the case of overexpression of the *lys* gene in *E. coli*. In the native host of HP1 phage, i.e., during *H. influenzae* infection, the participation of wild type HP1 holin protein is indispensable for lysis induction and phage progeny liberation took place only in the presence of HP1 holin. These in vivo studies suggested that holin may regulate the endolysin activity, since the interruption of the *hol* gene is sufficient to inhibit the cell lysis and phage release in *H. influenzae* cells. Moreover, the release of β-galactosidase was 1.875 times weaker in holin and endolysin expressing *E. coli* cells than in the case of expression of endolysin alone. This last result may be due to different expression levels of the *lys* gene alone in comparison to two overlapping *hol* and *lys* genes, but also may be due to a regulating role of HP1 holin in the endolysin-induced lysis process. Such role of pinholin in control of lysis was also described for ΦKMV phage [13].

We demonstrated that the holes formed by HP1 holin are too small to permit the leakage of large proteins like β-galactosidase (520 kDa) from *E. coli* cells. Similar observations were obtained in the case of S^21^68 pinholin of phage 21 using fluorescent periplasmic marker TorA-GFP-SsrA [2]. In contrast, lambda phage canonical holin supported the β-galactosidase flow from *E. coli* [2,33]. β-galactosidase leakage also took place in the case of the expression of canonical holin from *Actinomyces naeslundii* phage Av-1 in *E. coli* [28]. Membrane damage caused by HP1 holin seems to be insufficient to liberate the cell content, however sufficient to make impossible the preparation of spheroplasts. Observations under fluorescence microscope indicated that expression of the *hol* gene in *H. influenzae* Rd30 (induced by mitomycin C) was accompanied with increased mortality as compared to cells infected with mutant HP1 phage not expressing the holin. This phenomenon was already observed at the early stage of lysis of cells infected with wt HP1. In absence of *hol* expression, the infected *H. influenzae* cell shape was affected, probably by accumulation of new bacteriophages inside the cell (absence of cell lysis, confirmed by growing culture turbidity). Indeed, in Figure 5, we can see green points indicating a large amount of DNA, probably phage DNA in HP1Δ*hol*-lysogenized and induced *H. influenzae* cells. The addition of chloroform to *H. influenzae* infected with phage not expressing HP1 holin caused the membrane break down and liberated the progeny phage allowing the phage DNA extraction. This indicates that phage progeny was formed even in the absence of HP1 holin. Taking all these data together, HP1 holin rather does not cause lysis of the bacterial cell by itself (also confirmed by β-galactosidase assays and LIVE/DEAD staining), but seems to be indispensable to the lysis occurrence in vivo, which suggest its role in the control of the lytic process in native conditions. In *E. coli* cells, the overproduction of HP1 holin caused a dramatical decrease in cell viability, but not in turbidity of liquid culture. All the data together indicate that the product of HP1 *hol* gene could be a pinholin associated with a SAR-endolysin. 

In silico topological studies are not conclusive, indicating that HP1 holin may be a 2-TMD protein, with C-out topology or 1-TMD protein with N-out and C-in topology, as demonstrated in Figure 1. The well-studied S^21^68 pinholin has two TMD and reveals C-in, N-in topology [5]. In activated S^21^68, the first TMD is located in the cell membrane and the second TMD dimerizes to form holes: the activated S^21^68 is C-in, N-out [5]. The topology of other known pinholins is only predicted in silico and nothing is known about their active state topology. In our study, we prepared IMV from *E. coli* cells overexpressing the HP1 holin. With this strategy, we demonstrated that overexpressed HP1 pinholin has C-out and N-in topology. The preparation of spheroplasts, the structures deprived of the outer membrane and peptidoglycan, was impossible. We cannot exclude that overexpressed pinholin, embedded in the inner membrane in the active state, form holes that disrupt the inner membrane which, in turn, shows too many discontinuities to create a stable structure. Indeed, we demonstrated that the overexpression of HP1 pinholin in *E. coli* was insufficient to cause cell lysis (constant culture turbidity), but the cell membrane perturbation was sufficient to cause cell death (abrupt reduction of CFU/mL).

The C terminal domain of HP1 holin is much more hydrophilic than the rest of the protein, contains almost 50% charged amino acids, and is slightly positively charged (Figure 1). Usually, the positive-inside rule determines the topology of inner membrane proteins [34]. However, T4 phage holin, with long and highly hydrophilic C-terminus, has a C-out, N-in orientation [35]. The unusual periplasmic domain of T4 holin is involved in the control of lysis timing [36]. The T4 antiholin protein, analogous to the S107 antiholin of phage λ, blocks the lysis by binding the C-terminus of T4 holin. T4 holin has only one TMD and by tis is different of HP1 holin [7]. We hypothesize that the demonstrated unusual C-out and N-in topology of overexpressed HP1 pinholin reflects the active-lethal form of this protein i.e., the topology that forms holes after depolarization of the membrane (Figure 1). The nonactive HP1 holin would have the same topology that S^21^68 pinholin from phage 21, i.e., two TMD and C-in and N-in topology. Recently performed topological and phylogenetic analyses of phage holins and putative holins based on their average sizes, predicted topologies and organism source allowed to distinguish seven superfamilies comprising 52 families [24]. HP1 holin topology is characteristic for class II holins. However, the activation of S^21^68 consists in the exposure of N-terminal domain to the cytoplasm. In contrast, in the case of HP1 holin, the TMD2 and C-terminus would be externalized to the periplasm after its activation (Figure 1.). It is not clear what is the signal triggering the activation of HP1 holin. In contrary to the gene encoding S^21^68 pinholin from phage 21, the studied *hol* gene from HP1 phage does not possess a dual start motif. This excludes the existence of a sequence in HP1 lytic cassette encoding a protein comparable to S^21^71 antiholin. As we mentioned above, antiholin also regulates the activation of T4 phage holin with periplasmic C-terminus [36]. We screened all other genes encoded by HP1 phage for their similarity with the known protein, but none was similar to an antiholin. Possibly, the activation of lysis is the effect of the membrane depolarization as consequence of HP1 increased concentration in the inner membrane. Evaluation of the effect of HP1 lytic genes on β-galactosidase leakage suggested the control of lysis by holin (the leakage is inhibited in case of holin and endolysin co-expression in comparison to endolysin alone, but the mechanism remains unknown). Further studies should be performed to explain the exact mechanism of activation of HP1 holin and subsequent depolarization of the inner membrane.

## 4. Materials and Methods

### 4.1. Bacterial Strains, Phages, Plasmids, and Growth Conditions

*H. influenzae* Rd30 [37] and *H. influenzae* Rd30 lysogenized by wt HP1 or HP1Δ*hol* strains were grown in BHI (Becton, Dickinson, Difco, Wokingham, UK) supplemented with NAD and hemin (2 and 10 μg/mL, respectively) at 37 °C. *E. coli* ER1821 strain (purchased from NEB, Ipswich, MA, USA), *E. coli* Top 10 (ThermoFisherScientific, Invitrogen, Waltham, MA, USA), and *E. coli* strain BL21(DE3) (Sigma-Aldrich, Novagen, St. Louis, MO, USA) were grown in LB broth at 37 °C. If required, the media contained antibiotics or other supplements. For selection of kanamycin resistant *H. influenzae*, we used 7 µg/mL of kanamycin. *E. coli* carrying pMPMT4Ω and its variants with cloned genes were maintained in LB medium containing 10 μg/mL tetracycline. Cells carrying pET28a(+) and its derivatives with cloned genes were selected on LB medium with 30 μg/mL kanamycin and 25 μg/mL chloramphenicol. When indicated, sodium azide (NaN_3_) or CHCl_3_ were added to give final concentrations of 1 mM or 10%, respectively. Arabinose, glucose, IPTG, and X-gal were added at final concentrations of 1%, 1%, 0.5 mM and 40 μg/mL, respectively. For prophage induction, mitomycin C (Sigma-Aldrich) was added at the final concentration of 35 ng/mL. Plasmid pUC19 was purchased from Gibco-BRL (Rockville, MD, USA). pMPMT4Ω was originally obtained from M. Mayer [38], pET28a(+) was purchased from Sigma-Aldrich, Novagen.

### 4.2. Chemicals, Reagents, Enzymes, and Primers

Restriction and modifying enzymes and proteinase K were from Thermo Fisher Scientific (Waltham, MA, USA). All chemicals used for this study, reagent-grade or better, were from Sigma-Aldrich unless otherwise specified. LIVE/DEAD BacLight Bacterial Viability Kit was purchased from Thermo Fisher Scientific, Molecular Probes. His-tag Mouse Monoclonal Antibody and Goat Anti-Mouse IgG AP Conjugate were from Novagen. All primers used in this work are listed in Table 1 and were purchased in the DNA Sequencing and Oligonucleotide Synthesis Laboratory (oligo.pl) at the Institute of Biochemistry and Biophysics (IBB), Polish Academy of Sciences, Poland.

### 4.3. Cloning of Lytic System of HP1 Phage in E. coli

All general techniques were used according to protocols described for both host organisms—*E. coli* and *H. influenzae* [39,40].

The bacteriophage HP1 was originally obtained from R.M. Herriott. The DNA fragments carrying the potential phage HP1 *hol*, *lys*, or both genes on single fragment were amplified using primers PPLE and PLPH, PLLS and PLPS, PLPH, and PLPS, respectively, using HP1 DNA as template (Table 1). All amplicons (320, 619, and 918 bp long) were cloned into pMPMT4Ω vector [38], at EcoRI and HindIII or EcoRI and PstI sites, respectively, resulting in the formation of H (pMPMT4::*hol*), E (pMPMT4::*lys*), and HE (pMPMT4::*hollys*) plasmids. In these constructs, cloned genes were under the control of pBad promotor and may be expressed in both *E. coli* Top10 or *E. coli* ER1821 cells.

The *hol* gene was also cloned in pET28a(+) plasmid in such a way that the produced Hol protein was His-tagged on N-terminus (from construct pNHisHol) or on C-terminus (from construct pCHisHol). For construction of these plasmids, we used the PCR products obtained with primers HNHolL, HNHolR (HisTag added to the N-terminus of HP1 holin) and HCHolL, HCHolR (HisTag added to the C-terminus of HP1 holin) (Table 1).

The accuracy of all obtained constructs was verified by sequencing, performed in the DNA Sequencing and Oligonucleotide Synthesis Laboratory (oligo.pl) at the IBB.

### 4.4. Expression of Cloned HP1 Lysis Genes in E. coli

*E. coli* Top10 cells carrying constructs H, E, and HE mentioned above were cultivated overnight in liquid medium with antibiotics and glucose to stop the pBad promoter. Then, cultures were refreshed by inoculating 50 mL of LB with 0.5 mL of overnight-culture. Bacteria were grown until the culture turbidity reached OD_600_ about 0.45 and 1% arabinose was added to selected cultures to induce the expression of cloned genes. Samples were taken every 30 min, OD_600_ was measured and cells were plated after appropriate dilutions on LB containing plates.

To express His-tagged holin, a single colony generated by fresh transformation of *E. coli* BL21(DE3) carrying pNHisHol or pCHisHol construct was used to start a culture at 37 °C with intensive shaking (200 rpm). When cell culture entered logarithmic grow (OD_600_ about 0.5), IPTG was added to a final concentration of 0.5 mM and the cells were incubated for additional 45 min. Cells were then collected by centrifugation (15 min, 4 °C, 3000× *g*) and the pellet was used for further preparation of spheroplasts or inverted membrane vesicles, as described below.

### 4.5. Construction of Bacteriophage HP1 Deficient in hol Gene (HP1Δhol)

Phage HP1 with mutation within the *hol* gene (HP1Δ*hol*) was constructed by insertion of kanamycin-resistance cassette (*km*) from the pDIY-km plasmid [41] within the *hol* gene. Mutation was performed by transformation of *H. influenzae* cells, lysogenized by HP1 phage, with linearized construct pHol::*km* by Eco0109I. For transformation of *H. influenzae*, we used the starvation medium method (MIV) [40]. The pHol::*km* was constructed on the basis of pMPMT4::*hollys* plasmid with the *km* gene inserted within the *hol* gene. This vector contains DNA uptake sequences specific for *H. influenzae* and the sequences flanking the *hol::km* gene that were longer than 500 bp. The linearized construct was transformed into *H. influenzae* Rd30 (HP1) cells during the MIV procedure. The linearization of transforming DNA forces the occurrence of double crossing over and replacement of the wild-type *hol* gene by *hol::km* construct within the HP1 prophage, present in *H. influenzae* Rd30 cells. *H. influenzae hol*-deficient colonies (*hol::km* mutants) were selected on BHI plates containing kanamycin. With MAPHL and MAPHR primers (Table 1), we performed PCR on genomic DNA isolated from *H. influenzae* (HP1) cells resistant to kanamycin, and for further studies, we chose cells from which we obtained only one PCR product of 1197 bp, corresponding to the *hol* gene interrupted by *km* cassette and not a product of 315 bp corresponding to the wild-type *hol* gene. The PCR product was sequenced to prove the disruption of the *hol* gene within prophage HP1 genome.

### 4.6. H. influenzae Transformation

Briefly, *H. influenzae* Rd30 (HP1) cells were incubated for 2 h in BHI supplemented with NAD and hemin at 37 °C with intensive shaking (180 rpm). Then, bacteria were washed with no growth starvation MIV medium [40] and then cultivated in this medium for additional 1.5 h at 37 °C with gentle shaking (100 rpm). After this time, the linearized pHol::*km* was added to competent bacteria [42]. After 1 h of incubation with transforming DNA, cells were plated on BHI with NAD and hemin and kanamycin. Plates were incubated at 37 °C for 48 h.

### 4.7. Fluorescent Cell Staining and Microscope Observation

*H. influenzae* cells lysogenized by wild-type HP1 or *hol*-deficient HP1 (*hol::km*) were grown with standard conditions described above until OD_600_ reached 0.4. After induction of bacteriophages with mitomycin C for 3 h, cells were pelleted and rinsed with water and suspended in 2% formaldehyde. The optical density OD_600_ was adjusted to 0.03 and cells were stained with LIVE/DEAD BacLight Bacterial Viability Kit and deposed on filters XF25 (Omega Optical Inc., Brattleboro, VT, USA). Stained bacteria were observed with Nikon Eclipse E400 (Nikon, Amsterdam, Netherlands) with magnitude 400×. The number of dead and live cells was evaluated using ImageJ [27].

### 4.8. Phage DNA Extraction

Supernatants of *H. influenzae* (wt HP1 or HP1Δ*hol*) cultures induced by mitomicyn C were centrifuged twice (5 min, 13,000 rpm) in order to remove unlysed cells and cell debris. DNAse (Sigma-Aldrich) and RNAse (Sigma-Aldrich) were then added at concentration 1 and 10 μg/mL, respectively, for digestion of released bacterial nucleic acids. Phage particles were then concentrated by PEG method [39]. Phages were suspended in TE buffer and decapsidated by adding 1% SDS for 5 min at 68 °C. After supplementing with 100 mM final NaCl, DNA was extracted in phenol:TE buffer (twice) and chloroform:TE buffer. Then, phage DNA was precipitated with isopropanol and the pellet was washed with 70% ethanol and air-dried [39]. Phage DNA was resuspended in TE buffer and DNA yield checked by OD_260_ measurement (NanoDrop ND-1000, Thermo Fisher Scientific) and agarose gel electrophoresis.

### 4.9. Extracellular β-Galactosidase Activity Assay

Refreshed liquid cultures of *E. coli* ER1821 (expressing native β-galactosidase) cells carrying plasmids pMPMT4Ω, pMPMT4::*hol*, pMPMT4::*lys*, or pMPMT4::*hollys* were treated with 0.5 mM IPTG for induction of native, intracellular β-galactosidase expression. When OD_600_ reached 0.5, cultures were supplemented or not with 1% arabinose in order to induce the lytic cloned genes expression. After 1 h, the hydrolysis of *o*-nitrophenyl (ONP)-β-D-galactopyranoside at 28 °C and pH 7.0, followed by measurement of absorbance at 420 nm, was used for determination of extracellular β-galactosidase activity in culture supernatants. Enzyme activity was expressed in Miller units [43].

### 4.10. Preparation of Spheroplasts and Removing of Amino Acid Residues from Their Surface

Spheroplasts were prepared according to Bläsi et al. [44]. The pellets from *E. coli* cells, with IPTG-induced expression of His-tagged holin on N or C terminus, were suspended in spheroplasting buffer (40% sucrose; 20 mM EDTA; 60 mM Tris-HCl; pH 8) and lysozyme (10 μg/mL) was added. After 15 min of incubation in ice-bath, warm water (30 °C) was added and the incubation was continued at 30 °C. Cells were then supplemented with 20 mM MgSO_4_ and centrifuged. The pellet was resuspended in spheroplasting buffer supplemented with 20 mM MgSO_4_. The proteinase K was then added to the final concentration of 250 μg/mL to one sample and the reaction was performed for 1 h at 37 °C. The reaction was halted by adding PMSF (2 mM final concentration) and spheroplasts were collected by pelleting. The pellet was suspended in spheroplast lysing buffer (150 mM NaCl; 1 mM EDTA; 2% Triton X-100; 50 mM Tris-HCl; pH 8.0) and then ultracentrifuged (1 h, 100,000× *g*, 18 °C). From the obtained pellet, membrane proteins were extracted.

### 4.11. Preparation of Inverted Membrane Vesicles (IMV) and Removing of Amino Acid Residues from Their Surface

IMV were prepared as described by Bläsi et al. [44]. The pellets from *E. coli* cells, with IPTG-induced expression of His-tagged holin on N or C terminus, were suspended in buffer for cell disintegration in French Press (250 mM saccharose; 20 mM EDTA; 60 mM Tris-Cl; pH 8,0). The suspension was disrupted by passage through a SLM-Aminco French press (AIE, Haverhill, MA, USA) at 16,000 psi, generating the IMV sample, which was freed of unlysed cells by centrifugation in a microfuge tube at 3000× *g* for 1 min. The proteinase K was then added (250 μg/mL) for 1 h at 37 °C to one sample. The reaction was then stopped by adding PMSF (2 mM final concentration) and the IMV were ultracentrifuged (1 h, 100,000× *g*, 18 °C). Membrane proteins were extracted from the obtained pellet.

### 4.12. Extraction of Membrane Proteins

Spheroplast or IMV pellet after ultracentrifugation was suspended in sonication buffer (0.5 M NaCl; 35 mM MgCl_2_; 1% Triton X-100; 10% glycerol; 20 mM Tris-Cl; pH 8.0) and sonicated. The samples were then utracentrifuged again (1 h, 100,000× *g*, 18 °C), and pellet resuspended in membrane protein extraction buffer (35 mM MgCl_2_; 1% Triton X-100; 10 mM Tris-HCl; pH 8.0) for 12–16 h with intensive stirring at 37 °C. The samples were ultracentrifuged again (1 h, 100,000 × *g*, 18 °C), and resuspended in appropriate buffer (150 mM NaCl; 1 mM EDTA; 2% Triton X-100; 50 mM Tris-HCl; pH 8.0).

### 4.13. Western Blot Analysis of Extracted Membrane Proteins

Extracted membrane proteins from spheroplasts or IMV were separated on 16% Tricine-SDS-PAGE. Proteins were then transferred to PVDF (Sigma-Aldrich, Millipore) in a semi-dry blot procedure (overnight, 30 mA). The membrane was blocked with 1% Alkali-Soluble Casein (Sigma-Aldrich, Novagen) in TBS buffer (10 mM Tris-Cl; pH 7.5; 150 mM NaCl) for 1 h and washed twice in TBS. Then the membrane was incubated with primary anti-His-tag antibody (1:1000 in blocking solution, 60 min) and washed with TBSTT buffer (20 mM Tris-Cl; pH 7.5; 500 mM NaCl; 0.2% Triton X-100; 0.05% Tween 20) and TBS buffer. Then, the membrane was incubated with secondary goat-anti-mouse-IgG coupled to alkaline phosphatase (1:5000 in blocking solution, 60 min). After extensive washing with TBSTT buffer, the alkaline phosphatase was detected with chromogenic substrate BCIP/NBT according to the manufacturer’s protocol (Sigma-Aldrich).

### 4.14. Bioinformatic Analysis

The DNA and protein sequences were compared with the GenBank and SWISS-PROT databases on the BLAST server hosted by the National Center for Biotechnology Information. Protein subcellular localization was predicted by PSORTb [20]. The trans-membrane helices were identified with the help of the TMHMM 2.0 computer program (https://services.healthtech.dtu.dk/service.php?TMHMM-2.0), HMMTOP [45], TMpred [46], PHOBIUS (http://phobius.sbc.su.se/), DeepSig [22], Phyre2 [23], and SOSUI [21]. The presence of signal peptide sequences were investigated by SignalP 5.0 [26], HMMER [19], and iPSORT [47].

## 5. Conclusions

In conclusion, we characterized for the first time the HP1 bacteriophage lytic cassette that encodes at least two proteins that exhibit all the characteristics expected for a pinholin and SAR-endolysin combination. However, it seems that the lytic system of HP1 differs in some respect from such systems encoded by other tailed phages i.e., unusual exposure of C-terminus of holin to periplasm, when activated, absence of the known antiholin and perhaps in the way the holin activates.

## Figures and Tables

**Table 1:** Primer sequences.

Primer Name	Sequence 5′-3′	Used for
PLLE PLPH	CGGGAATTCATGAGTAAAAAATTTGGTGCA GTCAAGCTTTTTTACTCATCTATTGCTCC	Amplification of *hol* from HP1 genome (320 bp)
PLLS PLPS	CATGAATTCACTGTGCTGACGTATAACAAT GTACTGCAGAATACAGCCTAAAAACACAAT	Amplification of *lys* from HP1 genome (619 bp)
PLPH PLPS	GTCAAGCTTTTTTACTCATCTATTGCTCC GTACTGCAGAATACAGCCTAAAAACACAAT	Amplification of *hol lys* from HP1 genome (918 bp)
HNHolL HNHolR	TGGCTAGCTGTATGAATAGCAAAATAGATAGCGCA CGCAAGCTTTTACTCATCTATTGCTATCCCTA	Addition of HisTag at N-terminus of holin
HCHolL HCHolR	GACCATGGATGAATAGCAAAATAGATAGCGCA GCTCGAGACACTCATCTATTGCTATCCCTA	Addition of HisTag at C-terminus of holin

## Abbreviations

CFU	colony-forming units
IMV	inverted membrane vesicles
ORF	open reading frame
PFU	plaque-forming units
SAR	signal-arrest-release
TMD	transmembrane domain
